# Supplementary material for: Higher Levels of Early Childhood Caries (ECC) Is Associated with Developing Psychomotor Deficiency: The Cross- Sectional Bi-Township Analysis for The New Hypothesis
Source: Int J Environ Res Public Health. 2019 Aug 24;16(17):3082. doi: 10.3390/ijerph16173082 (PMC6747446; doi:10.3390/ijerph16173082)
Supplement: Supplementary file 1 [file ijerph-16-03082-s001.pdf]

Table S1. The CCDI measured in kindergarteners with different dmft scores detected.

	**Classification of dmft**																	
	**≦0**	**≧1**	***p***	**≦1**	**≧2**	***p***	**≦2**	**≧3**	***p***	**≦3**	**≧4**	***p***	**≦4**	**≧5**	**p**	**≦5**	≧6	*p*
Gross motor	72.66	81.82	0.0196 *	78.49	81.52	0.3354	80.83	81.19	0.8928	79.70	81.65	0.4511	80.53	81.39	0.7255	80.43	81.54	0.6326
Fine motor	98.03	96.57	0.7330	99.57	96.24	0.3215	100.81	95.62	0.0286 *	99.52	95.63	0.0891	99.69	95.28	0.0512	98.73	95.42	0.1373
Expressive language	90.95	90.55	0.9229	95.01	89.90	0.1179	95.74	89.25	0.0108 *	93.91	89.36	0.0690	93.71	89.13	0.0539	92.55	89.38	0.1651
Comprehension-concept	103.93	107.81	0.4116	108.59	107.36	0.7395	111.93	106.40	0.0732	111.41	106.12	0.0611	111.41	105.75	0.0349 *	111.53	105.12	0.0126 *
Situation comprehension	87.34	88.49	0.8024	90.32	88.12	0.5433	92.75	87.29	0.0579	89.33	88.06	0.6473	90.31	87.52	0.2865	89.66	87.63	0.4207
Self-help	91.30	90.91	0.9250	94.22	90.45	0.2405	95.50	89.79	0.0331 *	93.90	89.87	0.0979	94.20	89.45	0.0403*	92.94	89.74	0.1506
Personal-social	87.86	87.11	0.8544	89.42	86.82	0.4168	91.50	86.05	0.0284 *	90.94	85.81	0.0272 *	89.18	86.25	0.2088	88.41	86.42	0.3751
General development scale	100.16	102.10	0.5932	103.39	101.74	0.5610	104.98	101.18	0.1113	103.54	101.37	0.3184	103.81	101.10	0.1887	103.72	100.89	0.1524

	classification of dmft																	
	≦6	≧7	*p*	≦7	≧8	*p*	≦8	≧9	*p*	≦9	≧10	*p*	≦10	≧11	*p*	≦11	≧12	*p*
Gross motor	80.95	81.24	0.8993	81.26	80.96	0.8962	81.26	80.90	0.8793	82.15	79.00	0.2081	81.73	79.47	0.3964	81.13	81.03	0.9717
Fine motor	99.64	94.27	0.0157 *	99.55	93.68	0.0094 *	98.41	94.23	0.0688	98.55	93.01	0.0291 *	97.98	93.31	0.0896	97.52	93.70	0.1610
Expressive language	92.94	88.66	0.0545	92.59	88.47	0.0625	91.72	88.98	0.2218	92.09	87.65	0.0574	91.64	87.87	0.1257	91.16	88.54	0.3264
Comprehension-concept	112.62	103.48	0.0002 *	111.37	103.45	0.0014 *	109.83	104.20	0.0261 *	109.26	104.06	0.0481 *	108.94	103.80	0.0641	108.49	104.06	0.1416
Situation comprehension	90.31	86.86	0.1595	90.40	86.30	0.0925	89.71	86.55	0.2021	89.89	85.49	0.0873	89.55	85.44	0.1307	89.36	85.01	0.1718
Self-help	93.00	89.29	0.0876	92.77	89.01	0.0807	92.09	89.30	0.2036	92.16	88.53	0.1114	92.11	87.90	0.0798	91.98	87.23	0.0678
Personal-social	88.05	86.46	0.4656	87.95	86.35	0.4603	87.26	87.03	0.9148	87.69	86.13	0.4951	87.34	86.71	0.7931	87.32	86.61	0.7870
General development scale	104.41	99.98	0.0210*	104.17	99.61	0.0169*	103.17	100.20	0.1267	103.21	99.45	0.0638	102.88	99.55	0.1196	102.65	99.45	0.1667

	classification of dmft																	
	≦12	≧13	*p*	≦13	≧14	*p*	≦14	≧15	*p*	≦15	≧16	*p*	≦16	≧17	*p*	≦17	≧18	*p*
Gross motor	80.47	84.21	0.2219	80.73	83.83	0.3789	80.99	82.58	0.7133	80.94	83.47	0.5858	81.15	80.35	0.8895	81.20	78.34	0.6649
Fine motor	96.77	96.26	0.8648	97.01	94.45	0.4449	96.88	94.42	0.5500	96.65	97.07	0.9227	96.73	95.61	0.8315	96.60	98.83	0.7201
Expressive language	90.70	90.01	0.8106	90.71	89.68	0.7531	90.94	86.64	0.2835	90.91	86.35	0.2845	91.03	81.70	0.0695	90.63	89.09	0.7997
Comprehension-concept	107.98	105.37	0.4226	107.78	105.73	0.5800	108.00	102.10	0.1936	107.95	101.97	0.2153	107.72	103.51	0.4718	107.53	107.07	0.9461
Situation comprehension	88.52	87.86	0.8362	88.69	86.51	0.5466	88.82	83.77	0.2544	88.62	85.62	0.5241	88.48	86.88	0.7791	88.19	94.52	0.3440
Self-help	91.59	87.97	0.1998	91.40	87.88	0.2724	91.49	84.79	0.0877	91.51	83.58	0.0568	91.38	82.28	0.0710	91.13	85.79	0.3690
Personal-social	87.11	87.43	0.9082	87.38	85.76	0.6120	87.42	84.35	0.4324	87.32	85.23	0.6156	87.39	82.81	0.4707	87.17	87.12	0.9939
General development scale	102.12	101.16	0.6993	102.19	100.39	0.5279	102.20	99.18	0.3873	102.16	99.25	0.4332	102.11	98.88	0.4718	101.86	104.65	0.5968

*: $p < 0.05$.
